# Editorial: The integration of clinical veterinary anatomy and diagnostic imaging

**DOI:** 10.3389/fvets.2025.1602545

**Published:** 2025-05-20

**Authors:** Sokol Duro, Ozan Gündemir, Tomasz Szara

**Affiliations:** ^1^Department of Morphofunctional Modules, Faculty of Veterinary Medicine, Agricultural University, Tirana, Albania; ^2^Department of Anatomy, Faculty of Veterinary Medicine, Istanbul University—Cerrahpaşa, Istanbul, Türkiye; ^3^Department of Morphological Sciences, Institute of Veterinary Medicine, Warsaw University of Life Sciences, Warsaw, Poland

**Keywords:** anatomical structures, diagnostic imaging, innovative MRI techniques, spectral CT, shear wave elastography, vascular anatomy

*The integration of clinical veterinary anatomy and diagnostic imaging* has significantly advanced veterinary medicine, enhancing diagnostic precision, surgical planning, and therapeutic strategies. Veterinary anatomy provides the fundamental framework for understanding the structure and function of animal bodies. At the same time, imaging modalities offer non-invasive tools to visualize these structures in both normal and pathological states. The synergy between these fields has improved the accuracy of disease diagnosis and paved the way for more refined interventional procedures. With continuous advancements in imaging technologies, including higher-resolution imaging, three-dimensional reconstructions, and quantitative analysis, veterinary medicine is experiencing an unprecedented transformation. The Research Topic, “*The integration of clinical veterinary anatomy and diagnostic imaging*,” presents a collection of 14 studies that exemplify this interdisciplinary approach, contributing to a deeper understanding of anatomical variations, pathological conditions, and novel diagnostic methodologies.

The evolution of imaging technologies has been instrumental in veterinary diagnostics. Techniques such as computed tomography (CT), magnetic resonance imaging (MRI), and ultrasonography have become indispensable tools, providing detailed anatomical insights that facilitate the identification of pathologies challenging to detect through traditional methods. These modalities have expanded the diagnostic capabilities of veterinarians, allowing for early disease detection, improved treatment monitoring, and more precise therapeutic interventions. Several studies within this Research Topic highlight the practical applications of integrating anatomy and imaging:

Magnetic resonance imaging (MRI) is an exceptionally valuable tool in veterinary medicine, providing detailed visualization of musculoskeletal, nervous, and lymphatic structures. Several studies in this collection explore innovative MRI techniques and their potential applications in diagnosing and monitoring various conditions. They offer new approaches for early detection, disease characterization, and improved treatment planning.

Bunzendahl et al. investigated the magnetic properties of canine menisci using *ex vivo* MRI techniques to explore age-related joint degeneration. Their study assessed magnetization transfer, T1, and T2^*^ relaxation times as potential cartilage and meniscal health biomarkers. By analyzing meniscal tissue's structural and biochemical composition, the researchers identified patterns associated with degenerative changes, which could serve as early indicators of osteoarthritis. Their findings provide a foundation for developing non-invasive diagnostic techniques that allow veterinarians to track joint deterioration over time. The study's results emphasize the potential of MRI-based biomarkers in guiding therapeutic interventions to preserve joint function and mitigate osteoarthritic progression in aging dogs.

Lee et al. applied two-point and six-point Dixon MRI for fat fraction analysis in healthy dogs' lumbar vertebral bodies and paraspinal muscles, comparing the results with magnetic resonance spectroscopy. The study demonstrated that Dixon MRI provides a reliable and non-invasive method for quantifying fat infiltration in muscle tissue. It is a valuable tool for assessing muscle health and detecting early signs of atrophy. Their research suggests that this imaging technique could be beneficial for monitoring metabolic disorders and neuromuscular conditions that affect muscle composition. By eliminating the need for invasive biopsies, Dixon MRI offers a practical alternative for routine veterinary assessments and long-term health monitoring. The study further validates Dixon MRI as a faster, clinically feasible alternative to traditional magnetic resonance spectroscopy, increasing its applicability in veterinary practice.

DuPont and Boudreau measured canine medial retropharyngeal lymph nodes using T2 spin-echo sequences at 3T MRI to establish baseline values for normal lymph node size and texture. The study provides critical reference data for distinguishing between physiological variations and pathological enlargement due to lymphoma, infection, or metastatic disease. By defining normal morphometric parameters, this research improves the diagnostic utility of MRI in clinical settings, allowing for more accurate assessments of lymphadenopathy. Their findings highlight the importance of advanced imaging techniques in early disease detection, ultimately aiding in timely therapeutic decision-making. The study also explores the potential correlation between lymph node size and systemic disease, further expanding the role of MRI in clinical veterinary oncology.

Kim et al. detailed the MRI characteristics of an atypical cervical glioma with a predominant extramedullary distribution in a dog. The case study underscored the importance of MRI in differentiating spinal tumors based on their location, extent, and tissue characteristics, which directly influence treatment planning. Their imaging findings provided crucial insights into the compressive effects of the tumor on surrounding neural structures, guiding the surgical approach and postoperative management. The study further demonstrated the potential for significant neurological recovery following the intervention, even in severe spinal cord compression cases, emphasizing the importance of early and accurate imaging for optimizing treatment outcomes. By combining advanced MRI findings with histopathological confirmation, this case highlights the evolving role of MRI in diagnosing and managing complex spinal tumors in veterinary patients.

In addition to MRI, CT remains a highly effective tool in veterinary diagnostic imaging. By utilizing advanced computed tomography (CT) modalities—including spectral CT, cone beam CT (CBCT), multidetector CT (MDCT), and CT angiography—researchers are enhancing diagnostic accuracy, refining treatment strategies, and improving non-invasive assessment techniques. These innovations provide a more precise understanding of anatomical and pathological variations, ultimately optimizing clinical decision-making in veterinary medicine.

Ji et al. examined the echocardiographic and CT features of congenital bronchoesophageal artery hypertrophy and fistula in a dog. Their study highlighted the complementary roles of echocardiography and CT angiography in diagnosing complex vascular anomalies, which are often challenging to detect using a single imaging modality. A 4-year-old beagle underwent routine medical screening, revealing a right-sided continuous murmur. Further imaging identified multiple systemic-to-pulmonary shunts, which were surgically ligated. Postoperative imaging confirmed reduced ventricular overload and decreased shunt flow. This study provides valuable insight into the imaging features and surgical management of multi-origin systemic-to-pulmonary shunts, emphasizing the role of multimodal imaging in diagnosis and intervention.

Umh et al. conducted a detailed morphologic study of PDA in 25 dogs using computed tomography imaging, providing an in-depth characterization of anatomical variations in this congenital defect. By evaluating differences in PDA morphology, the study helps refine surgical and catheter-based closure techniques, ensuring better procedural outcomes. Their findings revealed three distinct PDA morphologies, with significant correlations between anatomical dimensions and body weight. The distinctive cross-sectional configuration observed via CT imaging offers valuable pre-procedural planning insights, potentially aiding the design of new occlusion devices. This research enhances interventional cardiology approaches, allowing for more tailored and effective treatment strategies.

Hagenbach et al. compared cone beam computed tomography (CBCT) with multidetector computed tomography (MDCT) for imaging the equine carpal region. Their study demonstrated that CBCT provides detailed visualization of bone and soft tissue structures with reduced radiation exposure compared to MDCT. In a study of 28 forelimbs from 15 horses, CBCT was effective for assessing osseous structures and some intraarticular ligaments, particularly after contrast enhancement. However, MDCT provided superior imaging of soft tissue structures and cartilage. These findings position CBCT as a reliable diagnostic tool for equine orthopedic evaluations, particularly useful in detecting early-stage musculoskeletal injuries such as stress fractures and osteoarthritis.

Mikić et al. evaluated virtual non-contrast spectral CT images compared to accurate unenhanced images in rabbits, demonstrating that spectral CT offers a viable alternative to conventional imaging with potentially lower radiation doses. By analyzing 219 regions of interest in 20 rabbits, the study confirmed that attenuation values between virtual and accurate non-contrast images were highly comparable, particularly in the spleen, liver, musculature, and renal cortices. Additionally, the elimination of motion artifacts and reduction in radiation exposure make this method particularly beneficial for fragile or repeatedly imaged patients. While the technique has shown promise for normal tissues, further research is needed to validate its applicability in diseased organs, paving the way for broader veterinary applications.

Shear Wave Elastography (SWE) has emerged as a valuable non-invasive diagnostic tool for assessing reproductive organ health in veterinary medicine. Zappone et al. investigated testicular stiffness in healthy and fertile male dogs using qualitative (2D-SWE) and quantitative (pSWE, 2D-SWE) techniques. Their study established baseline values for normal testicular stiffness in medium-to-large breed dogs, finding relatively uniform stiffness with minor variations across anatomical regions but no significant differences between testes, breeds, or age groups. Additionally, body weight showed a correlation with stiffness in 2D-SWE measurements. These findings highlight the potential of SWE for detecting early testicular abnormalities, improving reproductive management, and aiding in diagnosing subclinical infertility in breeding dogs. Further research with larger and more diverse canine populations will help refine its clinical applications.

Similarly, Uçmak et al. explored the use of SWE in monitoring postpartum reproductive organ recovery in Kivircik ewes. Their study quantified shear wave speed (SWS) and stiffness in the uterine cervix, caruncles, and vulvar labia, alongside Power Doppler ultrasonography to assess blood flow changes in caruncles. They observed significant time-dependent differences in cervical stiffness and caruncular regression, providing key insights into uterine involution. The findings demonstrate the potential of SWE in tracking postpartum recovery and fertility status in livestock, offering a new non-invasive approach to reproductive health assessment in ewes.

Together, these studies underscore the growing role of elastography in veterinary reproductive medicine. By enabling precise, real-time evaluation of tissue stiffness in different reproductive structures, SWE is promising to improve breeding management, early disease detection, and overall reproductive efficiency in companion animals and livestock.

Kleiner et al. and Wolf et al. investigated advanced techniques for sacroiliac luxation repair in feline cadaveric models. Both studies utilized CT-based neuronavigation to enhance surgical accuracy and safety. Kleiner et al. compared fluoroscopy-controlled freehand drilling to computer-assisted navigation, demonstrating improved screw placement precision with neuronavigation. Similarly, Wolf et al. assessed the feasibility of minimally invasive computer-assisted drilling (MICA) compared to the traditional fluoroscopy-controlled approach. Their study highlighted a steep learning curve but showed that computer-assisted techniques reduced surgical inaccuracies over time, improving accuracy deviations from 4.2 mm to 0.9 mm after five procedures. Moreover, Wolf et al. introduced a new patient reference array placement method, which improved safety by preventing violations of vital structures. These findings emphasize the role of CT-based image guidance in orthopedic procedures, enhancing surgical precision while minimizing neurovascular risks.

Godlewska et al. presented a case report on a nodular lesion in the ventral neck of a rat, utilizing radiography, ultrasound, and echocardiography alongside histopathology to establish a differential diagnosis. The study highlighted the challenges of assessing subcutaneous tumors in small mammals due to their rapid growth and varied etiologies. Their comprehensive multimodal imaging approach facilitated a more accurate lesion characterization, enabling targeted biopsy and subsequent therapeutic planning. The case emphasizes the importance of combining different imaging modalities in diagnosing rare or ambiguous masses in exotic animal practice.

Reinitz et al. utilized CT-based angiography with a barium contrast agent to generate a 3D reconstruction of the vascular anatomy in an African elephant's hindfoot. This study provides crucial insights into the unique blood supply to the digital cushion, supporting in the diagnosis, treatment, and prevention of foot diseases, which are a leading cause of morbidity in captive and wild elephants.

In conclusion, this Research Topic of studies underscores the pivotal role of advanced imaging techniques in modern veterinary medicine. By integrating detailed anatomical knowledge with cutting-edge imaging modalities, veterinary professionals can achieve more accurate diagnoses, refine surgical techniques, and improve patient outcomes across various species and conditions. The studies within this Research Topic illustrate how imaging is essential for diagnosing diseases and advancing anatomical knowledge, surgical precision, and therapeutic approaches in veterinary science.

